# Transcriptome analysis revealed chimeric RNAs, single nucleotide polymorphisms and allele-specific expression in porcine prenatal skeletal muscle

**DOI:** 10.1038/srep29039

**Published:** 2016-06-29

**Authors:** Yalan Yang, Zhonglin Tang, Xinhao Fan, Kui Xu, Yulian Mu, Rong Zhou, Kui Li

**Affiliations:** 1 The State Key Laboratory for Animal Nutrition, Institute of Animal Science, Chinese Academy of Agricultural Sciences, Beijing 100193, P.R.China; 2 Agricultural Genome Institute at Shenzhen, Chinese Academy of Agricultural Sciences, Shenzhen, 518124, P.R.China

## Abstract

Prenatal skeletal muscle development genetically determines postnatal muscle characteristics such as growth and meat quality in pigs. However, the molecular mechanisms underlying prenatal skeletal muscle development remain unclear. Here, we performed the first genome-wide analysis of chimeric RNAs, single nuclear polymorphisms (SNPs) and allele-specific expression (ASE) in prenatal skeletal muscle in pigs. We identified 14,810 protein coding genes and 163 high-confidence chimeric RNAs expressed in prenatal skeletal muscle. More than 94.5% of the chimeric RNAs obeyed the canonical GT/AG splice rule and were trans-splicing events. Ten and two RNAs were aligned to human and mouse chimeric transcripts, respectively. We detected 106,457 high-quality SNPs (6,955 novel), which were mostly (89.09%) located within QTLs for production traits. The high proportion of non-exonic SNPs revealed the incomplete annotation status of the current swine reference genome. ASE analysis revealed that 11,300 heterozygous SNPs showed allelic imbalance, whereas 131 ASE variants were located in the chimeric RNAs. Moreover, 4 ASE variants were associated with various economically relevant traits of pigs. Taken together, our data provide a source for studies of chimeric RNAs and biomarkers for pig breeding, while illuminating the complex transcriptional events underlying prenatal skeletal muscle development in mammals.

Muscle fibers are the basic structural and functional units of skeletal muscle^1^. The number of muscle fibers determines the capacity for postnatal muscle fiber growth^2^,^3^. Porcine skeletal muscle development is a complex biological process, especially during prenatal developmental stages. All muscle fibers are formed during the prenatal stage, whereas postnatal skeletal muscle development is mainly associated with increased muscle fiber size^4^. In pigs, prenatal myogenesis exhibits two major waves of fiber generation: primary fiber formation at 35–60 days post coitus (dpc) and secondary myogenesis at 54–90 dpc^5^. The majority of muscle fibers are formed during secondary myogenesis using the primary fibers as templates^6^. Previous studies showed that the critical time point for the formation of secondary myogenesis fibers was at approximately 63 dpc^7^, whereas the stages ranging from 49 to 77 dpc were pivotal for formation of various muscle phenotypes^8^. However, the molecular mechanisms underyling myofiber formation in mammals such as pigs remain unclear. Transcriptome profiling of prenatal skeletal muscle is an effective strategy for understanding the molecular events mediating myogenesis in pigs.

Gene expression profiles during tissue and organ development are complex. Multiple transcript types, including long non-coding RNA, chimeric RNA, and circular RNA, as well as transcriptional events, including alternative splicing and allele-specific expression (ASE), contribute to the complexity of the transcriptome and provide significant obstacles to the achievement of a comprehensive understanding of the genetic basis of skeletal muscle development^9^,^10^. Transcriptomic research on porcine skeletal muscle has mainly focused on mRNA^7^,^8^,^11^, miRNA^12^,^13^,^14^,^15^,^16^, and lncRNA^17^. No report exists regarding chimeric RNA, single nucleotide polymorphisms (SNPs), and allele-specific expression analysis in pig skeletal muscle.

Chimeric RNA molecules, also known as fusion transcripts, are composed of exons from two genes located at different genomic loci^18^,^19^. In the human genome, at least 4–5% of tandem genes are occasionally transcribed into chimeric proteins, suggesting that chimeric RNAs production is a common event with the potential to generate hundreds of additional proteins^20^. The presence of chimeric RNAs augments the number of transcriptional events and complexity of a given genome. Chimeric RNAs are suspected to function in cancer cells^21^,^22^, as well as in normal cells and tissues^18^,^23^,^24^. In a recent study, we identified a set of chimeric RNAs in pigs^19^. To our knowledge, our report was the first study on chimeric RNAs in mammalian skeletal muscle.

Biomarkers and information regarding allele-specific expression (ASE) associated with muscle growth are important in animal breeding. SNPs are the most abundant type of DNA sequence polymorphism and serve as powerful genetic markers in pig breeding^25^,^26^,^27^,^28^. A well-known example of a porcine SNP is the nonconservative R200Q substitution mutation in the protein kinase, AMP-activated, gamma 3 non-catalytic subunit (*PRKAG3*) gene, which is associated with high glycogen content in pig skeletal muscle^29^. ASE analysis is used to detect allelic imbalance in transcription and assess *cis*-regulatory variation^30^,^31^. At least 30% of genes are influenced by to ASE, which has a considerable impact on gene expression^32^. The RNA-seq approach provides an effective method for comprehensively identifying SNPs and ASE variants in transcribed regions of the genome.

In this study, we used high-throughput transcriptome sequencing to systematically explore transcriptional events associated with prenatal skeletal muscle development in pigs. We first carried out systematic identification and characterization of protein coding genes and chimeric RNAs. Subsequently, we analyzed SNPs and ASE in prenatal skeletal muscle of Tongcheng pigs. This study provides a resource of chimeric RNAs, SNPs, and ASE that illuminates the molecular events underlying prenatal porcine skeletal muscle development and allows the development of molecular markers for pig breeding.

## Results and Discussion

### Global expression analysis of protein coding genes in prenatal skeletal muscle

**Figure 1:**
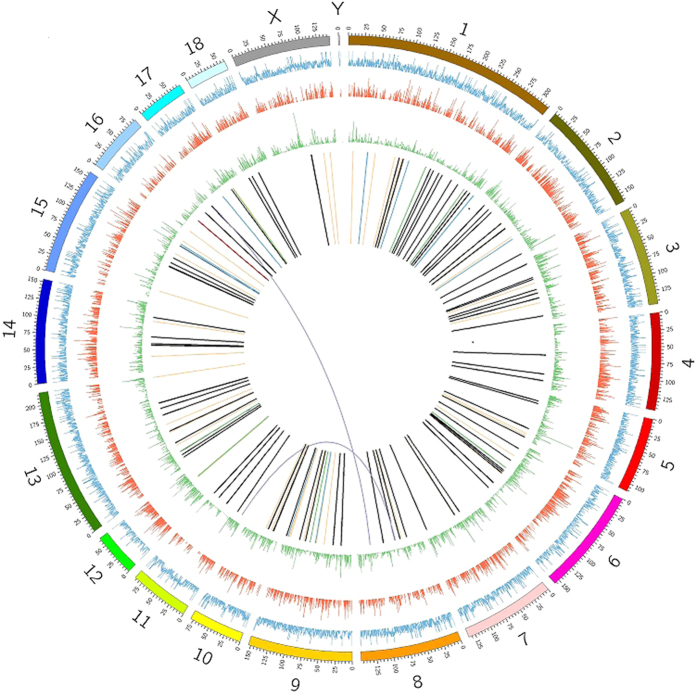
Transcriptome sequencing in prenatal porcine skeletal muscle. Chromosome ideograms are shown in the outer layer. The transcriptome sequencing coverage is shown in the first middle layer. Expression levels of genes are shown in the second middle layer. The SNP distribution is shown in the third middle layer. Chimeric RNAs are shown in the central layer. The chimeric RNA ssc-chimeric-113 is shown in red.

**Figure 2:**
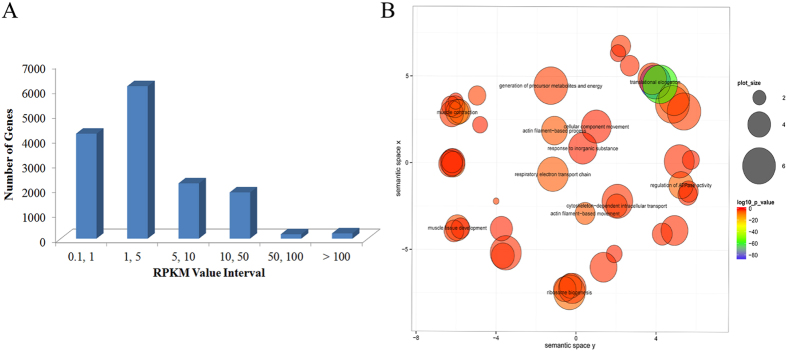
Analysis of protein coding genes in prenatal porcine skeletal muscle. (**A**) Distribution of detected protein coding genes with different expression levels. (**B**) GO biological process categories of the 200 most highly expressed genes.

Samples of prenatal skeletal muscle from Tongcheng pigs were analyzed using RNA-seq with a paired-end sequencing strategy on an Illumina HiSeq 2000 instrument. A total of 55.02 million 90-bp pair-end high-quality reads were obtained, of which 83.9% were mapped to *Sus scrofa* genome assembly 10.2. RPKM values were calculated to allow measurement of the expression levels of protein coding genes. Using RPKM >0.1 as a threshold, we detected 14,810 protein coding genes (PCGs) (Table S1), accounting for 68.54% of the PCGs included in the Ensembl release 78 mart database, indicating that most known PCGs were expressed in prenatal porcine skeletal muscle, while confirming that RNA-seq was an effective method for identifying PCGs with low expression levels. The read coverage of the RNA-seq data and the expression levels of the PCGs in the *Sus scrofa* reference genome are shown in Fig. 1. The PCG expression distribution is shown in Fig. 2A. In prenatal skeletal muscle, 69.9% of PCGs (10,347/14,810) were weakly expressed with RPKM <5, while only 1.4% (211/14,810) of PGCs were abundantly expressed with RPKM ≥100. Additionally, 25 highly expressed PCGs with RPKM values greater than 1000 were detected (Table 1). Gene ontology (GO) analysis of the 200 PCGs with the greatest transcript abundance revealed that genes associated with muscle development and contraction, such as *ACTC1*, *TNNC2*, *ACTA1*, *TNNC1*, *MYL3*, *ACTA2*, *MYH3*, and *MYL1*, were significantly enriched as expected; this phenomenon could be explained by the formation of the majority of muscle fibers during secondary myogenesis^6^. Genes involved in translational elongation (*EEF1G*, *EEF1B2*, *EEF2*, *EIF4G2*), ribosome biogenesis (*RPS* and *RPL* family genes), and regulation of ATPase activity (*NDUFA4*, *ND4L*, *NDUFB10*, *COX3*, *ND5*, *ND2*, *ND3*, *CYTB*, *ATP6*), which play essential roles in protein synthesis and fulfilling the energy requirements of prenatal skeletal muscle development, were significantly enriched (Fig. 2B). Two widely used housekeeping genes, β-actin (*ACTB*) and glyceraldehyde-3-phosphate dehydrogenase (*GAPDH*)^33^, were also highly expressed in prenatal skeletal muscle.

### Chimeric RNAs expressed in prenatal skeletal muscle

**Figure 3:**
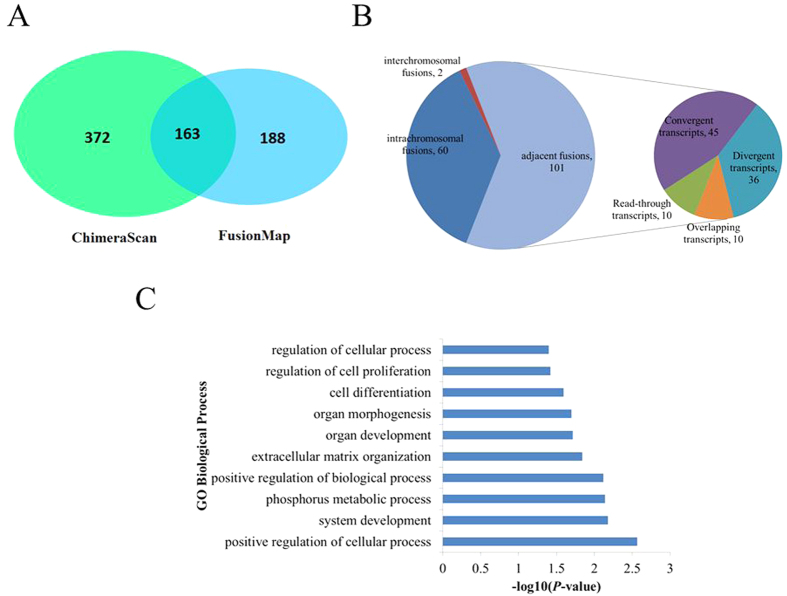
Identification of chimeric RNAs in prenatal porcine skeletal muscle. (**A**) Numbers of chimeric RNAs identified by ChimeraScan and FusionMap. (**B**) Classification of chimeric RNAs. (**C**) GO biological process analysis of the parental genes of chimeric RNAs.

Based on our transcriptome sequencing data, we identified chimeric RNAs associated with prenatal skeletal muscle development using the ChimeraScan^34^ and FusionMap^35^ programs. We detected 535 and 351 potential chimeric RNAs (including 163 RNAs detected by both programs) using ChimeraScan (Table S2) and FusionMap (Table S3), respectively (Fig. 3A). Of the 163 chimeric RNAs detected by both programs, 36.8% (n = 60) were intrachromosomal fusions, 62.0% (n = 101) were adjacent fusions, and only 1.2% (n = 2) were interchromosomal fusions (Fig. 3B). According to a previous study^36^, we classified the 101 adjacent fusions into four categories: 10 read-through transcripts, 45 convergent transcripts, 36 divergent transcripts, and 10 overlapping transcripts (Fig. 3B). The distribution of chimeric RNAs in the *Sus scrofa* genome is shown in Fig. 1. We found that 94.5% (154/163) of chimeric RNAs had canonical splice sites and obeyed the GT/AG rule, implying that chimeric RNAs were mainly formed by trans-splicing and had properties similar to those of protein coding genes to some extent. Indeed, previous studies have demonstrated that chimeric RNAs have the potential to be translated into functional proteins^18^,^37^. GO analysis showed that the parental genes of the chimeric RNAs were mainly involved in regulation of cellular process, system development, positive regulation of biological process, cell differentiation, and regulation of cell proliferation (Fig. 3C). These findings suggest that the identified chimeric RNAs likely play important roles in prenatal porcine skeletal muscle development.

To determine whether homologues of the chimeric RNAs identified in the current study exist in other species, we aligned them to chimeric transcripts from the human, mouse, and fruit fly genomes in the ChiTaRS2.1 database^38^. The alignment sequences were retained only when at least 20 nt of either side of the fusion junction could be mapped. Unfortunately, we found that only 10 and 2 of the chimeric RNAs identified in pigs had homologues in the human and mouse transcriptomes, respectively (Table S4), while no chimeric RNA homologues were identified in the fruit fly transcriptome. These findings suggest that chimeric RNAs in pigs exhibit high species specificity.

### Validation of chimeric RNAs

To validate the reliability of the group of identified chimeric RNAs, we selected 29 chimeric RNAs for RT-PCR verification in the same prenatal porcine skeletal muscle used for RNA sequencing analysis. The primers were designed to span the fusion junction of the chimeric RNAs. The vast majority of selected chimeric RNAs (20/29) were amplified by RT-PCR and confirmed by direct sequencing (Figure S1, Table S5). The consistency of the RT-PCR and prediction results suggests that the group of identified chimeric RNAs is sufficiently reliable for further research.

**Figure 4:**
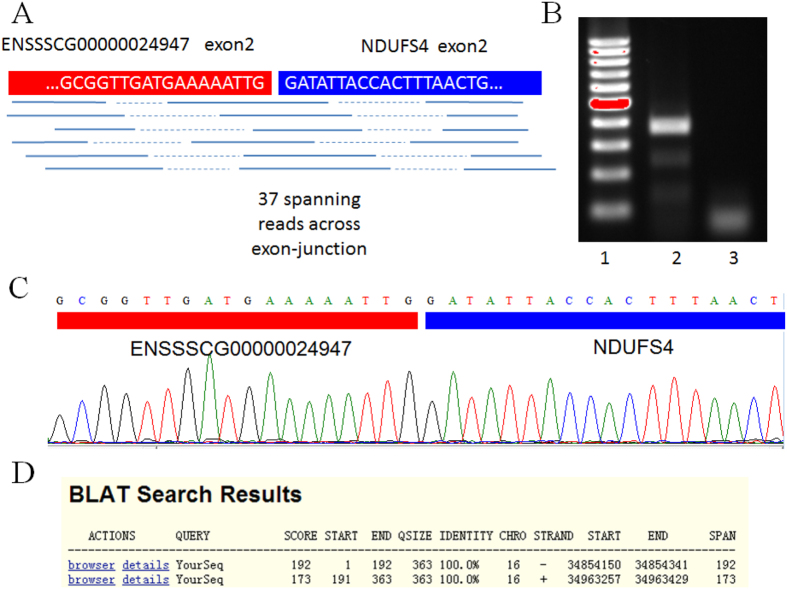
Validation of ssc-chimeric-113. (**A**) Detection of ssc-chimeric-113 via transcriptome sequencing. The “grep” command was used to identify 37 reads spanning the exon-junction. (**B**) Validation of ssc-chimeric-113 by PCR amplification and electrophoresis. Lane 1: marker. Lane 2: electrophoresis result. Lane 3: no template control. (**C**) Validation of the ssc-chimeric-113 breakpoint using Sanger sequencing. (**D**) BLAT of the Sanger sequencing result on *Sus scrofa* genome assembly 10.2 (http://genome.ucsc.edu/cgi-bin/hgBlat).

Subsequently, we focused on ssc-chimeric-113, a chimeric product generated from ENSSSCG00000024947 and *NDUFS4*. ssc-chimeric-113 was highly expressed in the results from the FusionMap (ranking 6^th^ with 151 seed counts) and ChimeraScan (ranking 8^th^ with a score of 139) analyses. The *NDUFS4* gene (NADH dehydrogenase (ubiquinone) Fe-S protein 4, 18kDa (NADH-coenzyme Q reductase)) is highly expressed in skeletal muscle and potentially related to intramuscular fat deposition in pigs^39^. A genome-wide association study (GWAS) showed that a single nucleotide polymorphism site in *NDUFS4* was significantly associated with loin muscle area^40^, implying that *NDUFS4* might play an important role in skeletal muscle development. The ENSSSCG00000024947 and *NDUFS4* genes are both located on chromosome 16, but on different strands. Our transcriptome sequencing data confirmed that ssc-chimeric-113 was abundantly expressed, as evidenced by 37 spanning reads across the fusion junction (Fig. 4A). This fusion junction was also confirmed using a dataset containing the transcriptome sequences of 9 different tissues in Guizhou pigs (data not shown). To verify the bioinformatics results, we performed PCR amplification of the prenatal skeletal muscle RNA used in transcriptome analysis, yielding a fragment 363 bp in length (Fig. 4B). Sanger sequencing showed that this PCR product was a fragment of ssc-chimeric-113 cDNA (Fig. 4C). BLAT of this sequence to *S. scrofa* genome assembly 10.2 showed that nucleotides 1–192 mapped onto the plus-strand of chromosome 16 at positions 34854150–34854341 in exon 2 of ENSSSCG00000024947, whereas nucleotides 191–363 mapped onto the minus-strand of chromosome 16 at positions 34963257–34963429 in exon 2 of *NDUFS4* (Fig. 4D). These results verified the existence of ssc-chimeric-113.

### Identifying SNPs in prenatal skeletal muscle

Whole-transcriptome RNA sequencing is an effective strategy for identifying polymorphisms in the genome, especially in transcribed regions. This approach has been used to identify candidate SNPs in exonic regions associated with traits of interest, including growth and meat quality^41^,^42^. To our knowledge, no such studies have been performed in porcine skeletal muscle at any developmental stage.

**Figure 5:**
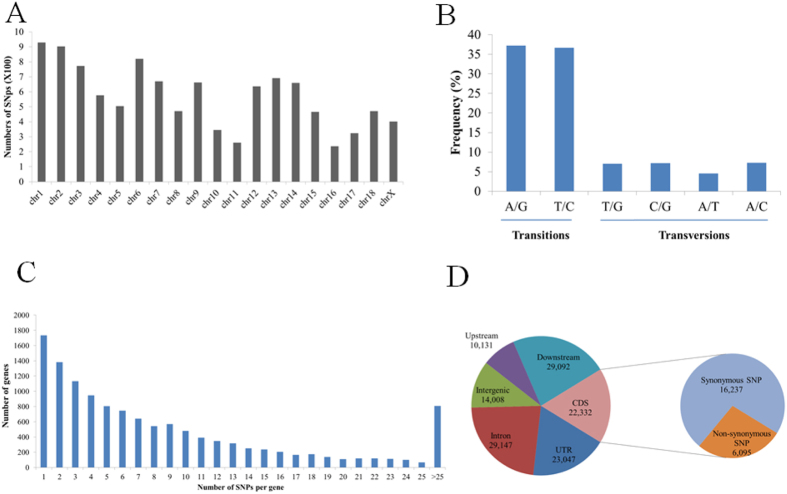
SNP identification in porcine prenatal skeletal muscle. (**A**) SNP distribution in porcine chromosomes. (**B**) Frequency of different substitution types in the identified SNPs. (**C**) Distribution of the number of SNPs per gene. (**D**) Distribution of SNPs in different genomic regions.

We identified 106,457 high quality SNPs in transcripts expressed in prenatal skeletal muscle (Table S6). The number of SNPs within each chromosome was directly proportional to chromosome length and gene number. Chromosome 1 contained the most SNPs, whereas chromosome 16 contained the fewest SNPs (Fig. 5A). The proportion of substitution transitions (A/G and C/T, 73.91%) was much higher than the proportion of transversions (A/C, A/T, G/C, and G/T; 26.09%). The frequency of A/G transition (37.2%) was similar to that of C/T transition (36.6%). Among transversions, the frequency of each type was approximately 7%, with the exception of A/G transition, for which the frequency was 4.6%. The transition:transversion ratio was 2.83:1 (Fig. 5B), which was similar to values reported in other species^41^,^43^. We found that 12,643 annotated genes contained one or more SNPs. The average number of SNPs per gene was 10.2, while 71.0% of genes had fewer than 10 SNPs. Interestingly, we found that 808 genes harbored more than 25 SNPs, implying that these genes exhibited high diversity. These results suggest that these 808 genes might be particularly susceptible to artificial selection and were helpful for understanding population diversity (Fig. 5C). We also compared the identified SNPs with the *S. scrofa* dbSNP database (Build 140); 93.6% of the variants (99,602 SNPs) were deposited in the dbSNP database, indicating the high quality and reliability of our SNP analysis. At the same time, we detected 6,955 novel SNPs. Our results have increased the number of known SNPs in *S. scrofa*.

### SNP annotation and function analysis

The distribution of the discovered SNPs within various genomic features was analyzed using Ensembl’s Variant Effect Predictor^44^. Of the SNPs present in coding regions, 6,095 were nonsynonymous, whereas 16,237 were synonymous. The ratio of nonsynonymous to synonymous SNPs was approximately 0.37 (6,095/16,237). We also identified 23,047 SNPs located at 5′- or 3′-UTR regions and 29,147 SNPs in intronic regions (Fig. 5D). In addition, we detected 26 SNPs in termination codons and 222 SNPs in splice sites, which may affect transcript splicing and thus potentially affect protein products and their functions (Table 2, Table S6). A large proportion of SNPs identified fell into the intronic and intergenic regions, providing evidence for the incomplete annotation status of the current swine reference genome and suggesting that comprehensive exploration of the transcriptome profiles of pigs is merited.

**Figure 6:**
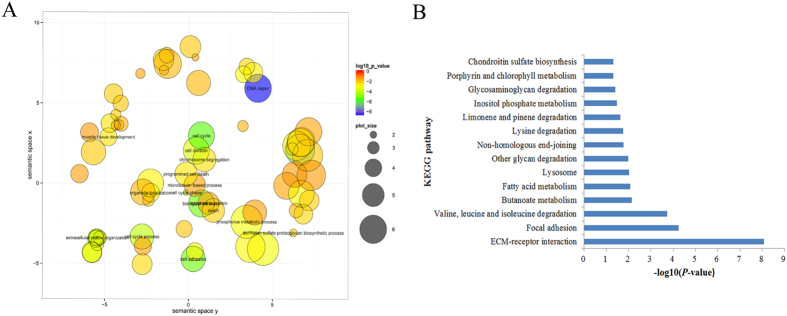
Functional annotation of genes containing nonsynonymous SNPs. (**A**) GO biological process analysis results. (**B**) KEGG pathway analysis.

Non-synonymous coding SNPs were further analyzed because they might result in amino acid substitution and thus affect protein activity. We carried out GO and KEGG enrichment analysis to investigate the putative functions of 1804 genes containing nonsynonymous SNPs. The results of these analyses revealed that 132 GO biological process terms were significantly enriched in the set of 1804 genes containing nonsynonymous SNPs (*p* < 0.05). These genes containing nonsynonymous SNPs were mainly involved in the response to DNA damage stimulus, DNA repair, the cellular response to stress, DNA metabolic processes, and the cell cycle (Fig. 6A). Interestingly, muscle development-related GO terms, including muscle cell development, skeletal muscle organ development, skeletal muscle tissue development, and muscle fiber development, were also significantly enriched in the set of genes containing nonsynonymous SNPs. This finding might be explained by the high expression levels of muscle development-related genes in prenatal skeletal muscle. These results demonstrate that our strategy is a powerful method of identifying SNP biomarkers associated with growth and meat quality traits. We found that a set of 641 genes containing nonsynonymous SNPs was significantly enriched in 14 KEGG pathways, including ECM-receptor interaction, focal adhesion, butanoate metabolism, and fatty acid metabolism (*p* < 0.01) (Fig. 6B). Moreover, we identified 1,046 SNPs in the set of chimeric RNAs, of which 988 SNPs (94.5%), including 95 nonsynonymous SNPs and 295 synonymous SNPs, were annotated in the dbSNP database and thus might be considered as candidate markers for studying the functions of chimeric RNAs in pigs.

Next, we queried the set of 106,457 high-quality SNPs to determine their presence in *S. scrofa* quantitative trait loci (QTLs) deposited in the AnimalQTLdb^45^. We counted the numbers of SNPs located in QTLs associated with production traits. There were 94,839 SNPs (89.09%) located within 685 QTL regions related to 90 production-related traits (Table S7). For example, 46,301 SNPs were located in 78 QTL regions for body weight at birth, whereas 45,809 SNPs were located in 181 QTL regions for average daily gain. The high proportion of SNPs located within QTLs for production-related traits indicates that our analysis is an effective strategy for detecting candidate quantitative trait nucleotides responsible for genetic variability influencing production traits.

### ASE analysis in prenatal skeletal muscle

**Figure 7:**
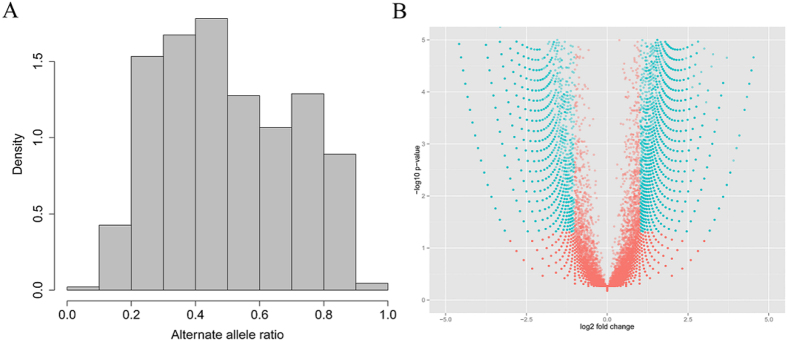
Allele-specific expression analysis of heterozygous SNPs in porcine prenatal skeletal muscle. (**A**) Distribution of the alternate allele ratio for all heterozygous sites expressed in prenatal porcine skeletal muscle. (**B**) Volcano plot analysis of heterozygous SNPs with allelic imbalance. The blue points are SNPs showing significant ASE, whereas red points are SNPs with no significant ASE.

Gene expression is influenced by *cis*- and *trans*-regulatory genetic variation. Genome-wide ASE analysis is an effective method for inferring the existence of *cis*-regulatory variants^30^,^46^. In this study, the ASEReadCounter tool was used to retrieve allele counts. Subsequently, a binomial test and Benjamini-Hochberg false discovery rate (FDR) correction were performed to identify ASE variants. The allelic distribution ratios, defined as the ratio of the abundance of the non-reference allele to the sum of the abundance of the non-reference allele and that of the reference allele, are shown in Fig. 7A. The analysis revealed that 11,300 heterozygous SNPs exhibited allelic imbalance (allelic ratios >0.65 or <0.35 and FDR <0.05) (Fig. 7B, Table S8), of which 845 SNPs were heterozygous-derived nonsynonymous variants, including 138 SNPs classified by Sift^47^ as “deleterious”. We then tested whether sites exhibiting ASE were more likely to be nonsynonymous SNPs, revealing a significant difference in the proportion of nonsynonymous SNPs with significant ASE and that of the entire set of analyzed SNPs (Fisher’s exact test, *p* < 0.001), which suggested an enrichment of nonsynonymous variants in ASE. In addition, we detected 131 ASE SNPs located in the chimeric RNAs.

GWASs have reported a large number of SNPs associated with phenotypes of various economic traits in pigs. To illuminate the functional impacts of SNPs, we examined whether SNPs reported by previous GWASs exhibited ASE in our study. Surprisingly, we identified 4 ASE variants reported by previous GWASs. Of these, SNP rs335265740 in the 3′-UTR of nuclear receptor subfamily 3, group C, member 1 (glucocorticoid receptor) (*NR3C1*) was associated with relative flare fat^48^. SNPs rs45433464 (located in stearoyl-CoA desaturase (delta-9-desaturase) (*SCD*)) and rs81215882 (located in phosphoglucomutase 1 (*PGM1*)) were significantly associated with average daily weight gain^49^,^50^. SNP rs80863153 in the 5′-UTR of aldehyde dehydrogenase 18 family, member A1 (*ALDH18A1*) was associated with hematological traits^51^. The analysis of allelic imbalance suggested that *cis*-regulatory variations might be associated with phenotypic divergence in pigs. Additionally, the rs340729607 (T/A) variant introduced a premature stop codon in exon 7 of mitochondrial ribosomal protein L1 (*MRPL1*), indicating nonsense-mediated decay. Unfortunately, we did not detect ASE variants in the chimeric RNAs generated from genes reported to influence economically important traits in the GWASs.

## Conclusion

In this study, we first performed a comprehensive analysis of chimeric RNAs, SNPs, and ASE variants in prenatal skeletal muscle using RNA-seq. We identified 163 high-confidence chimeric RNAs potentially associated with porcine prenatal skeletal muscle development. The existence of chimeric RNAs in pigs broadened our knowledge of the complexity of mammalian transcriptomes and illuminated the gene interaction network that functions during skeletal muscle development. The newly discovered SNPs and ASE variants expand the catalog of genetic variants in pigs and will facilitate molecular marker-assisted selection in pig breeding and relevant GWASs. This study provides a foundation for studies aimed at revealing the complex transcriptional mechanisms underlying prenatal skeletal muscle development in mammals, as well as a molecular marker resource that can be utilized in pig breeding. However, further studies are needed to decipher the biological functions of the chimeric RNAs, SNPs, and ASE variants identified in this study.

## Materials and Methods

### Animals and sample collection

All animal experiments were performed according to the procedures defined by national and local animal welfare bodies and were approved by the Institutional Animal Care and Use Committee at the Institute of Animal Science, Chinese Academy of Agricultural Sciences. The *longissimus dorsi* muscle samples were isolated from Tongcheng two pig fetuses (one male and one female) at 5 time points (gestational days 50, 55, 60, 65, and 75). All samples were maintained in liquid nitrogen until use.

### RNA extraction and high-throughput paired-end RNA-sequencing

Total RNA was extracted using Trizol (Invitrogen, Carlsbad, CA, USA) following the manufacturer’s protocol. RNA integrity was measured using an Agilent 2100 Bioanalyzer. Only samples with RNA Integrity Number (RIN) values greater than eight were used for sequencing. Library construction and Solexa sequencing were performed using methods described previously^17^ according to the manufacturer’s instructions (Illumina, USA). Briefly, total RNA from samples collected at five time points were pooled into a single sample in equal proportions. PolyA + RNA was purified from total RNA using magnetic oligo(dT) and fragmented. First-strand cDNA was generated using Random Primer p(dN)6 and Superscript III (Invitrogen, Carlsbad, CA, USA), after which second-strand cDNA synthesis and adaptor ligation were performed. cDNA fragments of 240–310 bp were isolated. The library was sequenced on the Illumina HiSeq 2000 platform to generate 90-bp paired-end reads.

### Transcriptome mapping and expression quantification

After filtering low quality reads, clean reads were mapped against the *S. scrofa* reference genome (assembly 10.2)^52^ using Tophat version 2.1.0^53^ with default options. Assignment of reads to genes was performed using htseq-count^54^. The expression levels of protein coding genes were measured as numbers of reads per kilobase of exon per gene per million mapped reads (RPKM)^55^.

### Identification of *Sus scrofa* chimeric RNAs

ChimeraScan (version 0.4.3)^34^ and FusionMap (version 2015-03-31)^35^ software was used to identify chimeric RNAs with the Ensembl release 78 reference genome (*S. scrofa* assembly 10.2)^52^ using default parameters. Classification of adjacent chimeric RNAs was performed as described in a previous study^36^: (1) read-through genes, adjacent genes in the same orientation; (2) diverging genes, adjacent genes in opposite orientations whose 5′ ends are in close proximity; (3) convergent genes, adjacent genes in opposite orientations whose 3′ ends are in close proximity; (4) overlapping genes, adjacent genes who share common exons. For conservation analysis of the pig identified chimeric RNAs, we downloaded sets of human, mouse, and fruit fly chimeric transcripts from the ChiTaRS 2.1 database^38^ and aligned the pig chimeric RNAs to those from other species using the BLAST program (Basic Local Alignment Search Tool, version 2.2.2 6+) with default parameters^56^ (at least 20 nt of the sequence on either side of the fusion junction must have been mapped). The “grep” command was used to search the reads spanning the fusion junction sequences of the ENSSSCG00000024947-NDUFS4 chimeric RNA from the fastq files of the transcriptome data as described previously^57^.

### Reverse transcription polymerase chain reaction

To validate the identified chimeric RNAs, total RNA from prenatal porcine skeletal muscle was reverse-transcribed into cDNA using the RevertAid First Strand cDNA Synthesis Kit (MBI Fermentas, Vilnius, Lithuania) according to the manufacturer’s protocols. The chimeric cDNA containing the fusion junction was amplified by PCR as follows: an initial denaturation at 94 °C for 3 min, followed by 34 cycles of denaturation at 95 °C for 15 s, annealing at 60 °C for 30 s, and elongation at 72 °C for 20 s, and a final extension for 5 min at 68 °C. The PCR products were confirmed by direct sequencing.

### SNP identification and annotation

The Genome Analysis Toolkit (GATK version 3.3) package^58^ was used for SNP discovery according to the best practice recommendations regarding the RNA-seq variant analysis workflow of the Broad Institute (https://www.broadinstitute.org/gatk/guide/best-practices?bpm=RNAseq). Stringent parameters were used to minimize detection of false-positive SNPs. Clusters of at least 3 SNPs within a window of 35 bases were filtered out. Hard filtering values, including Fisher strand values (FS > 30.0), qual by depth values (QD < 2.0), and read depth value (DP < 5), were selected. SNPs located on unplaced scaffolds and mitochondria were not included in this study. SNP annotation was performed using in-house Perl scripts and Ensembl’s Variant Effect Predictor^44^.

### ASE analysis

The ASEReadCounter tool^59^ in the GATK package was used to retrieve the allele counts of heterozygous SNP sites. Heterozygous sites with individual allele read depth less than 3 and total (both alleles) read depth less than 10 were filtered out. A binomial test and Benjamini-Hochberg FDR correction were performed. Cut-off criteria of allele ratio >0.65 or <0.35 and FDR <0.05 were used to identify significant allelic imbalances.

### Gene ontology and KEGG pathway analysis

Gene ontology (GO) and KEGG pathway enrichment analyses were performed with the Database for Annotation, Visualization, and Integrated Discovery (DAVID) website (http://david.abcc.ncifcrf.gov/)^60^. Because of the poor pig Ensembl annotations in the DAVID database, we converted the pig Ensembl gene IDs into human gene symbol IDs with Biomart (http://www.biomart.org/) before performing the GO and KEGG pathway analyses. We set the EASE value to 0.05 for the enrichment analysis. Significantly enriched GO biological process terms were summarized and visualized using REVIGO (http://revigo.irb.hr/)^61^.

## Additional Information

**Accession code**: The RNA-seq raw data from this study have been deposited in the NCBI Sequence Read Archive with accession number SRP066035. (http://www.ncbi.nlm.nih.gov/Traces/sra/).

**How to cite this article**: Yang, Y. *et al*. Transcriptome analysis revealed chimeric RNAs, single nucleotide polymorphisms and allele-specific expression in porcine prenatal skeletal muscle. *Sci. Rep.* **6**, 29039; doi: 10.1038/srep29039 (2016).

## Supplementary Material

Supplementary Information

Supplementary Data 1

Supplementary Data 2

Supplementary Data 3

Supplementary Data 4

Supplementary Data 5

Supplementary Data 6

Supplementary Data 7

Supplementary Data 8

## Figures and Tables

**Table 1:** The most highly expressed protein coding genes (RPKM >1000) in prenatal skeletal muscle.

**Ensembl Gene ID**	**Gene symbol**	**Description**	**RPKM**
ENSSSCG00000018082	MT-CO3	Cytochrome c oxidase subunit 3	7,133.76
ENSSSCG00000018075	MT-CO1	Cytochrome c oxidase subunit 1	6,221.94
ENSSSCG00000018081	ATP6	ATP synthase subunit a	5,381.04
ENSSSCG00000018078	MT-COII	Cytochrome c oxidase subunit 2	4,344.78
ENSSSCG00000021943			3,431.54
ENSSSCG00000004803	ACTC1	Actin, alpha, cardiac muscle 1	2,598.12
ENSSSCG00000004489	EEF1A	Eukaryotic translation elongation factor 1 alpha 1	2,469.99
ENSSSCG00000007799	HUMMLC2B	Myosin light chain, phosphorylatable, fast skeletal muscle	2,409.61
ENSSSCG00000017581	COL1A1	Collagen type I alpha 1	2,348.98
ENSSSCG00000016157	MYL1	Myosin light chain 1/3, skeletal muscle isoform	2,247.39
ENSSSCG00000018087	MT-ND4	NADH-ubiquinone oxidoreductase chain 4	2,207.78
ENSSSCG00000018094	CYTB	Cytochrome b	2,166.85
ENSSSCG00000007424	TNNC2	Cytochrome c oxidase subunit 1	1,824.34
ENSSSCG00000010190	ACTA1	Actin, alpha 1, skeletal muscle	1,821.53
ENSSSCG00000018084	ND3	NADH-ubiquinone oxidoreductase chain 3	1,686.45
ENSSSCG00000000694	GAPDH	Glyceraldehyde-3-phosphate dehydrogenase	1,423.94
ENSSSCG00000018065	MT-ND1	NADH-ubiquinone oxidoreductase chain 1	1,403.79
ENSSSCG00000014540	FTH1	Ferritin, heavy polypeptide 1	1,303.34
ENSSSCG00000018092	NADH6	NADH-ubiquinone oxidoreductase chain 6	1,189.82
ENSSSCG00000018069	MT-ND2	NADH-ubiquinone oxidoreductase chain 2	1,166.88
ENSSSCG00000006558	RPS27	Ribosomal protein S27	1,138.51
ENSSSCG00000025883	RPL37	Ribosomal protein L37	1,065.38
ENSSSCG00000005316	TPM2	Tropomyosin 2 (beta)	1,030.23
ENSSSCG00000005003	LOC100736624	40S ribosomal protein S29	1,026.09
ENSSSCG00000015103	RPS25	Ribosomal protein S25	1,022.35

**Table 2:** Annotation and classification of putative SNPs.

**SNP annotation**	**Number of SNPs**
Total SNPs	106,457
SNPs in annotated genes	12,643
UTR	23,047
Intron	29,147
Intergenic	14,008
Non-synonymous SNPs	6,095
Synonymous SNP	16,237
CDS	22,332
Splice region	222
Termination codons	26
Upstream	10,131
Downstream	29,092
