# Supplementary material for: Transcriptome analysis revealed chimeric RNAs, single nucleotide polymorphisms and allele-specific expression in porcine prenatal skeletal muscle
Source: Sci Rep. 2016 Jun 29;6:29039. doi: 10.1038/srep29039 (PMC4926253; doi:10.1038/srep29039)
Supplement: Supplementary Information [file srep29039-s1.doc]

**Supplementary Information for**

**Transcriptome analysis revealed chimeric RNAs, single nucleotide polymorphisms and allele-specific expression in porcine prenatal skeletal muscle**

Yalan Yang^1,2*^, Zhonglin Tang^1,2*^, Xinhao Fan^1^, Kui Xu^1^, Yulian Mu^1^, Rong Zhou^1^, Kui Li^1,2^

1. The State Key Laboratory for Animal Nutrition, Institute of Animal Science, Chinese Academy of Agricultural Sciences, Beijing 100193, P.R.China;
2. Agricultural Genome Institute at Shenzhen, Chinese Academy of Agricultural Sciences, Shenzhen, 518124, P.R.China.

^*^ These authors contributed equally to this work

Correspondence and requests for materials should be addressed to R.Z. (zhourong03@caas.cn) and K.L. (likui@caas.cn)

**Supporting information**

**Table S1.** RPKM values of protein coding genes in prenatal porcine skeletal muscle.

**Table S2.** Chimeric RNAs identified by ChimeraScan.

**Table S3.** Chimeric RNAs identified by FusionMap.

**Table S4.** Conservation analysis of pig chimeric RNAs across species. Pig chimeric mRNAs were aligned to known human and mouse chimeric transcripts deposited in the ChiTaRS 2.1 database.

**Table S5.** Primer information of selected chimeric RNAs for RT-PCR validation.

**Table S6.** Identification and annotation of SNPs in prenatal porcine skeletal muscle.

**Table S7.** List of SNPs located within QTLs for production traits.

**Table S8.** List of heterozygous SNPs which showing allelic imbalance.

**Figure S1.** Chimeric RNAs validated by RT-PCR. The chimeric RNAs corresponded to each line were listed in Table S5.


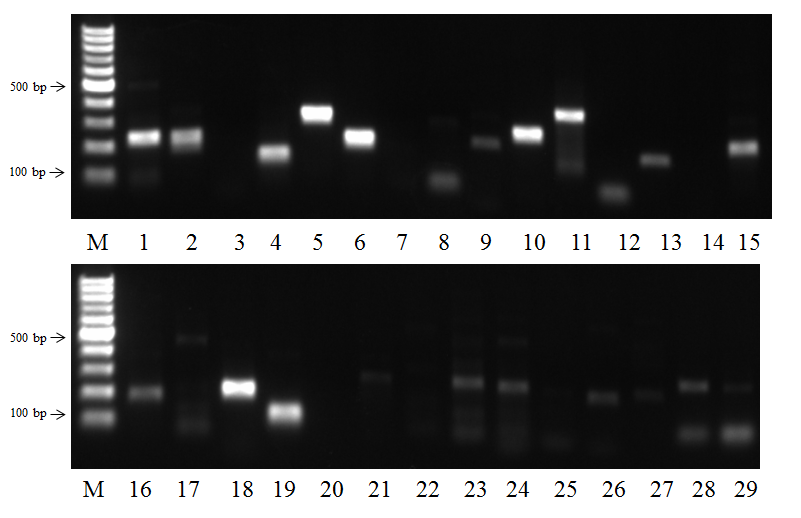


**Figure S1.** Chimeric RNAs validated by RT-PCR. The chimeric RNAs corresponded to each line were listed in Table S5.
